# Cardiovascular Ischemia: Advancement and Potential Use of Collagen-Based Therapeutic Strategies

**DOI:** 10.3390/ijms26199275

**Published:** 2025-09-23

**Authors:** Ayodeji A. Olabiyi, Lisandra E. de Castro Braz

**Affiliations:** Department of Physiology, Brody School of Medicine, East Carolina University, Greenville, NC 27858, USA; olabiyia22@ecu.edu

**Keywords:** collagen-based biomaterials, cardiac ischemia, remodeling

## Abstract

Cardiac ischemia causes a shortage of blood flow and oxygen to the heart muscle (i.e., myocardial infarction, MI), causing cell damage/death that initiates a cascade of healing processes. Every year, more than one million people in the United States die as a result of MI. After MI, approximately 40% of patients develop maladaptive myocardial remodeling that associates with decreasing cardiac function levels. Collagen treatment, particularly the use of collagen-based biomaterials as well as collagen supplementation, has shown promise in treating cardiac ischemia by supporting processes involved in blood pressure regulation, arterial health, and cholesterol management, mechanical support, increasing stem cell retention, and improving bioactive chemical delivery for myocardial repair and regeneration. In this review, we evaluate collagen treatment in myocardial ischemia based on existing studies and propose a missing link for future research.

## 1. Introduction

Cardiac ischemia (CI) refers to oxygen and nutrient deprivation to cardiac muscle that may lead to permanent cell damage and death, this is often caused by constricted coronary arteries. Although a blood clot or blood vessel constriction can induce narrowing, the most common cause is plaque formation, also known as atherosclerosis. Most persons with early CI (less than 50% coronary narrowing) do not feel any symptoms or restriction of blood flow. However, if the atherosclerosis advances, especially if left untreated, symptoms could arise. They are particularly likely to occur after activity or mental stress, when the demand for oxygen in the blood rises. Based on the global disease burden, it was estimated that CI affects roughly 126 million people (1655 per 100,000), or about 1.72% of the world’s population [1,2]. CI was responsible for nine million fatalities globally. Men are thought to be more commonly affected than women, and the prevalence usually begins in the fourth decade and rises with age [3]. The global prevalence of CI is increasing day by day. The prevalence, currently 1655 per 100,000, is expected to rise to 1845 by 2030 [1].

CI occurs when prolonged or severe reduction of blood flow to the heart restricts downstream oxygen and nutrient delivery, causing cell death. This can cause a considerable loss of cardiomyocytes, particularly if the patient is not reperfused; cell damage and/or death will lead to myocardial remodeling and poses significant obstacles to cardiac function recovery [4,5]. Because adult cardiomyocytes have limited regenerative capacity, the infarcted myocardium is replaced with scar tissue made primarily of extracellular matrix (ECM), which provides structural support but does not restore the heart’s functional capacity [6]. Cardiac ECM mostly comprises collagen and elastic fibers, and a variety of proteoglycans, glycoproteins, and glycosaminoglycans. The composition and ratio of the individual ECM components will change upon injury and scar formation. ECM remodeling, fibrosis formation, vascular changes, as well as cellular infiltration accompanied by reduced contractility, electrical remodeling, and ventricular remodeling are all changes that occur as a result of disease and can lead to long term cardiac adaptation. The cumulative effect of these modifications in cardiac composition causes changes in the structure, mechanics, and regulation of cellular responses in the heart.

Collagen is the primary component of ECM and possesses several optimal properties, such as biocompatibility, mechanical properties, porosity, degradability, low immunogenicity, and accessibility [7], making it an appropriate material to mimic cardiac ECM. Collagen-based biomaterials, thus, have the necessary characteristics to provide temporary mechanical support for ischemic tissue, to delay adverse remodeling processes, preserve cardiac function, and provide an ideal extracellular environment for the remaining myocardium [8]. While collagen is essential for healthy connective tissue throughout the body, including the arteries, its significance as a supplement in myocardial ischemia and heart health has not been systematically considered. This review aims to evaluate available collagen-based therapeutic strategies in CI, with particular emphasis on the roles of collagen supplementation and Collagen-based biomaterial applications, integrating current evidence to identify knowledge gaps and propose directions for future research.

## 2. Collagens in the Heart and Blood Vessels

Collagen is essential for the formation and function of the heart and blood vessels, acting as a scaffold for cardiomyocytes and vasculature while also contributing to blood vessel mechanical qualities [9,10] Therefore, collagens are important components of the ECM in the cardiovascular system, contributing to its integrity and stability. Collagen’s content and type are determined by a dynamic balance of synthesis and degradation [11]. Disruptions to the delicate balance of collagen synthesis and secretion, post-translational modifications, and degradation often have a negative impact on cardiac functionality.

According to their function and domain homology, collagens can be categorized into fibril-forming collagens, fibril-associated collagens with interrupted triple helices (FACITs), anchoring fibrils, network-forming collagens, transmembrane collagens, and beaded filament-forming collagens [12]. The arrangement of polypeptide chains and the variety of terminals distinguishes fibrillar and non-fibrillar collagens. Collagen types vary in conformation, resulting in various helical lengths and non-helical segment distributions. These parameters are used to classify collagens into various categories. About 30 different types of collagens have been identified in humans; some of the collagens present in different tissue types are shown in Table 1.

Fibrillar collagen is the most abundant protein in the cardiac ECM, while elastin fibers are present to a lower amount in the ventricular myocardium [27,28,29]. The majority of myocardial collagen fibers are made up of collagen types I and III, which account respectively for roughly 60% and 30% of collagen in the healthy heart [30,31]. These collagen fibers are intricately organized at the myocyte and myofibrillar bundle levels. The endomysial collagen network binds individual myocytes by Z-band-integrin connections; this conformation is essential to prevent ventricular dilatation by keeping cardiomyocytes aligned [32]. On the other hand, perimysial collagen surrounds complete myofibrillar bundles, typically forming a weave-like structure that gives tensile strength [33]. Thus, the fundamental functions of collagen in the heart are to provide a structural framework for cardiomyocytes, strengthen the myocardial wall, and aid in force transmission [34]. It is understandable that collagen synthesis, post-translational modification, and degradation are highly regulated processes, and even minor changes to the collagen matrix can have a significant impact on myocardial force development [35], relaxation and diastolic stiffness, and conduction properties that lead to arrhythmogenesis [36,37].

**Figure 1:**
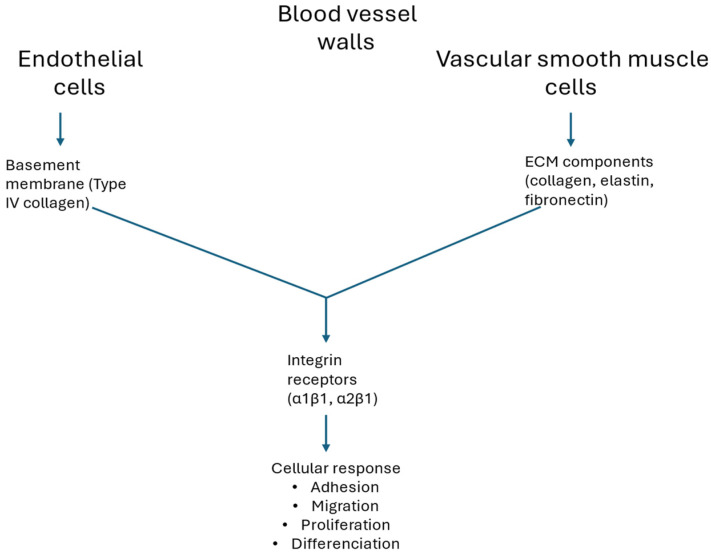
Collagen interactions with cells lining blood vessels.

Cardiac fibroblasts synthesize collagen as a procollagen molecule with amine and carboxy terminal pro-peptide domains. For collagen to be deposited as a mature fibril, a series of post-synthesis processing steps are performed in the extracellular space. This includes the cleavage of pro-peptides by particular enzymes [38], connection with matricellular proteins, and self-assembly of collagen molecules into staggered fibrils [39]. Collagen can be further stabilized by cross-linking, which occurs via at least two known mechanisms: lysyl oxidase (LOX)-mediated aldehyde formation between lysine or hydroxylysine residues [40], and advanced glycation end product (AGE) formation between amino groups by reducing sugars [41]. In the blood vessels collagen is a structural protein that is generally found lining the vessel inner walls. When a blood vessel is ruptured, collagen is exposed, attracting platelets, which results in blood clotting. Thus, collagen also has an important role in determining how cells lining the blood vessel walls function [40,42], especially endothelial cells, smooth muscle cells, and pericytes, which are essential for vascular structure, tone, and integrity (Figure 1). Aside from the structural role, collagen actively participates in signaling pathways that influence how these cells grow, move, and interact with their environment. Collagen functions in these capacities to provide structural support and guide ECM organization, cell adhesion and migration, regulation of endothelial cell behavior, influence on smooth muscle cells, angiogenesis, mechano-transduction, and pathological remodeling [43,44,45,46,47].

## 3. Collagen-Based Therapies for Cardiovascular Healing

Collagen-based therapies have emerged as a promising strategy in cardiovascular medicine [48], particularly for the repair and regeneration of heart tissue following ischemic injury, such as myocardial infarction (MI). As described above, given that collagen is a principal component of the cardiac ECM, it plays a critical role in wound healing, cell signaling, and mechanical support within the myocardium. Therefore, therapeutic approaches using collagen-based biomaterials aim to replicate or restore these native functions to promote tissue repair, reduce scar formation, and enhance angiogenesis.

Collagen-based biomaterials are used to repair damaged blood vessels and rebuild arteries [9] to seal blood vessels following procedures such as thoracic endovascular aortic repair (TEVAR) or endovascular aneurysm repair (EVAR), and as a scaffold or support matrix for tissue regeneration, especially in vascular tissue engineering [49]. Collagen, particularly types I and III, provides mechanical strength and elasticity to the heart. During injury or stress, the ECM undergoes significant remodeling, with an increase in collagen deposition, especially type I, which leads to fibrosis and ventricular stiffening [11]. Collagen-based interventions seek to modulate this remodeling process by supporting regeneration rather than scar formation. Injectable collagen hydrogels have been explored as minimally invasive delivery systems to the infarcted myocardium. These hydrogels can be engineered to match the mechanical properties of cardiac tissue and act as a supportive matrix for cell migration, angiogenesis, and paracrine signaling. For example, Yu et al. (2023a) [50] demonstrated that an in situ polymerizing collagen-based hydrogel injected into infarcted rat hearts promoted capillary density, reduced left ventricular dilation, and improved overall function. Similarly, Giraud et al. (2008) [51] reported that collagen–alginate hydrogels enhanced neovascularization and supported cardiomyocyte survival in ischemic tissue. Collagen scaffolds are also used as vehicles for controlled release of growth factors and therapeutic cells. They help deliver agents like vascular endothelial growth factor (VEGF), basic fibroblast growth factor (bFGF), or stem cells (e.g., MSCs or iPSCs) to the damaged myocardium in a sustained and localized manner, promoting angiogenesis and cardiac repair. For instance, [52] loaded bFGF into collagen matrices implanted onto infarcted myocardium, resulting in increased vascularization and functional recovery. The scaffold maintained local retention of the growth factor, which is often a challenge in systemic delivery.

Collagen-based cardiac patches, either acellular or cell-seeded, provide structural reinforcement to the infarcted wall and promote myocardial tissue regeneration. These patches can be sutured or adhered to the epicardial surface to deliver cells or biomolecules directly to the injury site. A study [53] developed a decellularized ECM hydrogel derived from porcine myocardium rich in collagen and ECM proteins. This hydrogel supported cardiac progenitor cell engraftment and enhanced functional recovery in animal models post-MI. Additionally, collagen–elastin patches, like Matriderm, have been adapted for cardiac applications due to their elasticity and angiogenic potential [54].

In addition to its role in cardiovascular disease, collagen therapy is used to treat several comorbidities such as diabetes mellitus, rheumatoid arthritis, osteoarthritis, osteoporosis, psoriatic arthritis, sarcopenia, gastric reflux, periodontitis, and skin wounds and/or aging. Collagen-based therapies have been shown to promote wound healing in a variety of injuries, for example, collagen dressings and supplements can aid in tissue recovery by supporting repair, angiogenesis, and fibrosis management [55]. This versatility makes collagen a viable option for managing different conditions, that affect distinct organs, and in the treatment of complex conditions. The various therapeutic uses of collagen, in the context of managing comorbidities, are highlighted by its functions in wound healing, musculoskeletal disorders, cardiovascular health, and gastrointestinal illnesses [56].

## 4. Sources and Types of Collagen-Derived Materials

The source and form of collagen significantly influence its biological properties and mechanical behavior both in clinical and experimental applications. There are two major sources of collagen-derived materials:

1. Animal-Derived Collagen: Bovine-derived collagen, primarily extracted from dermis or tendons, is one of the most widely used types in biomedical research and clinical practice. It offers a high yield of type I collagen, making it suitable for producing gels, films, and sponges. However, concerns about zoonotic disease transmission and immunogenic responses have led to strict purification and cross-linking processes [57]. Porcine skin and small intestinal submucosa (SIS) are also common sources, offering types I and III collagens. Porcine collagen is often used in wound dressings, dental membranes, and vascular grafts due to its similarity to human tissue structure [58]. Marine collagen, extracted from fish skin, scales, or jellyfish, is gaining attention due to lower disease transmission risk and religious/cultural acceptability. While marine collagen generally has lower thermal stability, it has proven useful in cosmetic, pharmaceutical, and biomedical applications, particularly in hydrogels and scaffolds for soft tissue engineering [59]. It is important to note that collagen’s structure and sequence are highly conserved across mammalian species, which is why collagen is generally biocompatible and has a low risk of immune rejection. For instance, the primary amino acid sequence of fibrillar collagens (especially type I and III, which are abundant in heart and blood vessels) is highly conserved between species such as bovine, porcine, and human [57,58]. Structurally, the triple-helical assembly of collagen (Gly–X–Y repeats, often Gly–Pro–Hyp) is a key determinant of its mechanical stability and is preserved across vertebrates. This structural conservation means that the epitopes recognized by immune cells are minimal or masked in the native triple helix. Nonetheless, even though collagen is highly conserved across species, it displays variations in its amino acid sequences that can create unique epitopes; these species-specific epitopes may trigger an immune response [60].
2. Recombinant and Synthetic Collagen: This collagen is produced using genetically engineered microorganisms (e.g., yeast, bacteria) to express human collagen genes. This approach eliminates risks associated with animal-derived products and allows for precise control of molecular composition. Recombinant collagens are still under development but show promise in ophthalmic, dermal, and cardiac applications [61].

**Figure 2:**
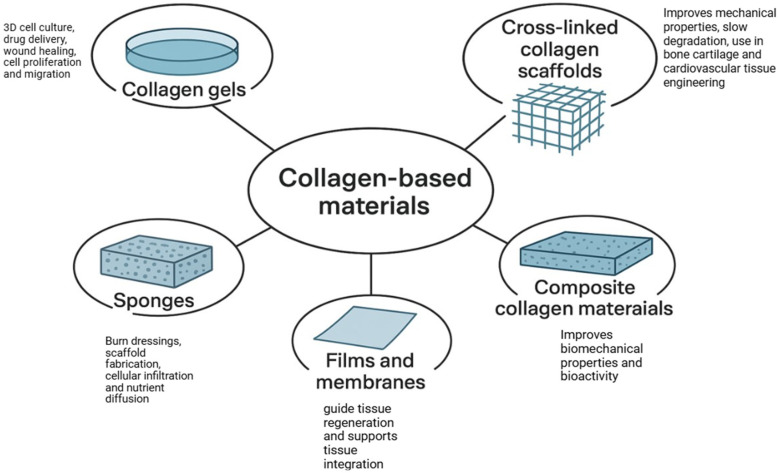
Different forms of collagen-based biomaterials and their applications (Illustration made using BioRender; https://BioRender.com/1us7oa1, accessed on 21 August 2025).

Independent of the source, collagen-based biomaterials exist in different forms which include collagen gels, sponges, films and membranes, cross-linked collagen scaffolds, and composite collagen materials (Figure 2). Collagen gels are typically used for 3D cell culture, drug delivery, and wound healing. They mimic native ECM structure and support cell proliferation and migration, though they have limited mechanical strength [62]. Produced by freeze-drying collagen suspensions, sponges are porous and widely used in hemostatic applications, burn dressings, and scaffold fabrication. Sponges’ porosity allows for excellent cellular infiltration and nutrient diffusion [63], while collagen films and membranes comprise flat, dense sheets commonly used in barrier membranes for guided tissue regeneration, especially in oral and maxillofacial surgery. They serve as resorbable barriers that support tissue integration. Thus, the formulation and format of the collagen-material is designed towards the biological and functional needs. Cross-linking (using agents like EDC (1-ethyl-3-(3-dimethylaminopropyl) carbodiimide hydrochloride) and NHS (N-hydroxysuccinimide), genipin, or UV light) improves mechanical properties and slows degradation. These scaffolds are used in bone, cartilage, infected areas, and cardiovascular tissue engineering [64].

Moreover, collagen is often blended with other materials (e.g., hydroxyapatite, elastin, alginate) to enhance functionality. These composites offer improved biomechanical properties and bioactivity tailored to specific applications [65].

The versatility of collagen-derived materials lies in their source diversity and structural adaptability. In summary, whether sourced from bovine, porcine, marine, or recombinant systems, collagen can be fabricated into gels, sponges, membranes, and composite scaffolds for a wide range of therapeutic uses (Figure 2). Ongoing advancements aim to optimize collagen’s mechanical integrity, biological performance, and immunological safety, ensuring its continued relevance in regenerative medicine.

## 5. Collagen-Based Materials in Support of Angiogenesis

Collagen serves as a scaffold for endothelial cell movement and organization during angiogenesis. Collagen is naturally recognized by the body and interacts with cells via integrins (e.g., α1β1, α2β1), discoidin domain receptors (DDRs), and other receptors [66]. These interactions stimulate endothelial cell adhesion, migration, and tube formation—critical steps in angiogenesis.

In addition, collagen-based materials are biodegradable, allowing gradual breakdown by matrix metalloproteinases (MMPs) that can be secreted by infiltrating cells. This degradation releases bioactive collagen fragments, some of which (e.g., proline-hydroxyproline) may further promote cell signaling and vessel growth [67,68].

VEGF plays a central role in orchestrating both new blood vessel formation and the synthesis of collagen, indicating a closely linked regulatory mechanism between angiogenesis and ECM remodeling. Research [69] demonstrated that effective angiogenic activity within a type I collagen environment (i.e., collagen from rat tail tendons cultured with human umbilical vein endothelial cells, HUVECs) relies on the engagement of α2β1 integrins with a specific sequence (GFP*GER) in the collagen molecule. This interaction is thought to activate intracellular signaling pathways, such as p38 MAP kinase, and promote the breakdown of focal adhesions, facilitating endothelial cell movement. These findings highlight the importance of integrin-collagen interactions and VEGF signaling in driving the cellular events required for blood vessel development. In this model, capillary morphogenesis happened before there was time for significant cell proliferation or migration, mimicking the tube creation stage of angiogenesis. While the model employed has some parallels to fibrin-induced angiogenesis and other sandwich-type angiogenesis systems, in which tubes form within 12–24 h, it differs from other 3-D collagen systems, in which angiogenesis normally occurs within 72 h.

Interestingly, when the apical gel was removed at an early stage, morphogenesis was inhibited, resulting in disassembly of the tube-like network, indicating that collagen must always be present for angiogenesis to occur. This finding was supported in another comparable model [70]. However, in cultures where the collagen gels were withdrawn after 8–10 h of HUVEC exposure, some intracellular gaps remained for up to 24 h, though a major fraction of the cells reverted to a monolayer [69]. A proposed explanation is that a provisional matrix is formed during morphogenesis, marking the intercellular gaps in the monolayer and preventing subsequent cellular repopulation. These findings offer a starting point for understanding the mechanism(s) behind type I collagen-driven angiogenesis. Additional research is needed to determine if α2β1 ligation of type I collagen activates p38 MAPK and enhances capillary tube formation. A report demonstrated that collagen fibers give guidance cues for capillary renewal during regenerative angiogenesis [71]. Collagens serve important roles in regenerative and developmental angiogenesis, according to in vivo and in vitro research, structural investigations, and manipulation of collagen production/cross-linking [59,72]. Collagen fibers (mostly type I collagen) promote and guide endothelial cell migration, while type IV collagen is important for appropriate lumen development and vascular integrity [72].

**Figure 3:**
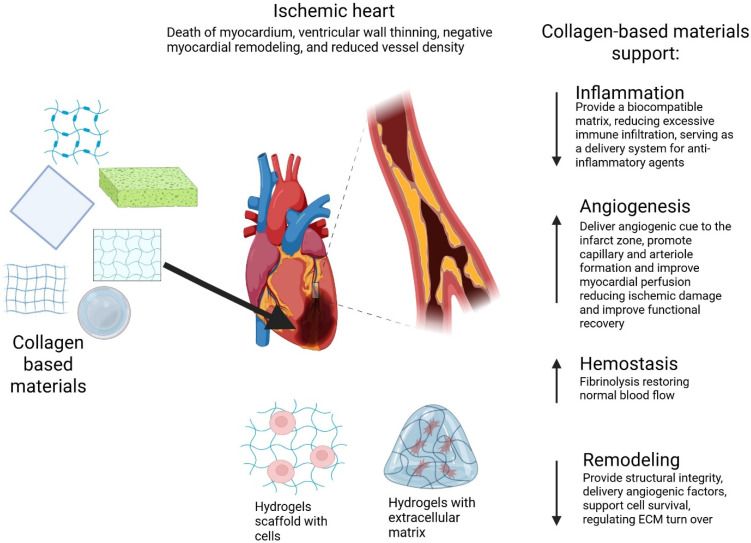
Schematic diagram illustrating the role of collagen in cardiac ischemia treatment (Illustration made using BioRender; https://BioRender.com/uvay1ga, accessed on 21 August 2025).

In general, changes in the levels of proangiogenic and antiangiogenic substances in the vasculature’s environment are hypothesized to influence the angiogenesis process. Numerous proangiogenic and antiangiogenic mediators have been identified. Proteins including bFGF, interleukin-8, platelet-derived growth factor, placental growth factor (PlGF), transforming growth factor-β, and VEGF promote angiogenesis (Figure 3), while angiostatin (plasminogen fragment), endostatin (type XVIII collagen fragment), and thrombospondin-1 inhibit it. In a quiescent state, the maintenance of vessels is thought to occur when the level of antiangiogenic signals outweighs the level of proangiogenic signals; however, periods of active angiogenesis occur when endothelial cells detect a shift in the balance of these mediators, with proangiogenic signals predominating [73].

## 6. Bioactive Collagen Materials in Support of Myocardial Repair

Collagen plays a central role in myocardial repair following injury such as myocardial infarction (MI), facilitating scar tissue formation necessary to restore structural integrity and support tissue regeneration [55,74]. Cardiac tissue healing progresses through four overlapping stages—hemostasis, inflammation, proliferation, and remodeling—each engaging various cell types [55] (Figure 3). During early phases, platelets initiate thrombogenesis, while activated endothelial cells and innate immune responses drive inflammation [75]. This leads to fibroblast recruitment and endothelial cell proliferation, producing granulation tissue enriched in proteoglycans and collagen [58].

As healing progresses, angiogenesis supplies oxygen and nutrients, while excess fibroblasts and vasculature undergo apoptosis to restore homeostasis. However, unresolved inflammation can lead to maladaptive remodeling and fibrosis. The extracellular matrix (ECM), particularly collagen, is central to this process, as it provides tensile strength and guides cellular behavior. Fibroblasts transition into myofibroblasts, secreting ECM proteins like type III and eventually type I collagen to stabilize the scar [76]. While sufficient ECM deposition is essential to prevent ventricular rupture, excess collagen may stiffen the myocardium, impairing diastolic function and promoting arrhythmias [77].

Cardiac fibrosis, characterized by excessive collagen deposition, is driven by complex signaling networks involving growth factors and cytokines. Myofibroblasts are central effectors, secreting ECM proteins, especially collagens I and III [11,78,79]. Type I collagen promotes fibroblast proliferation via ERK1/2 activation, whereas types III and VI have limited impact on this pathway [80]. However, type VI collagen supports myofibroblast differentiation. Interestingly, its upregulation post-MI and interaction with α-integrin receptors influence fibrosis, and its deletion reduces infarct size and fibrosis [81].

Collagen maturation, including cross-linking density, determines scar tensile strength and impacts cardiac function [82]. Type V collagen has emerged as a regulator of scar size, with Col5a1 deletion leading to abnormal scar architecture and increased fibroblast activation through mechanosensitive integrin signaling [83].

Given these multifaceted roles, collagen-based biomaterials are being actively explored for cardiac regeneration. Scaffolds, hydrogels, and composite materials provide structural support, promote angiogenesis, and modulate fibrosis (Figure 3). For example, Copes et al. (2019) [9] demonstrated that collagen scaffolds enhance stem cell retention, promote neovascularization, and improve myocardial function post-MI. Similarly, collagen hydrogels, due to their biocompatibility and water content, support stem cell survival and differentiation, reducing scar formation and enhancing cardiac function [51].

Hybrid collagen-based biomaterials offer additional benefits. Collagen–polymer composites, such as those combining collagen with poly(lactic-co-glycolic acid) (PLGA), show improved mechanical properties and controlled degradation [84,85]. The inclusion of glycosaminoglycans (e.g., heparin, hyaluronic acid) enhances cell infiltration and ECM remodeling [86]. A recent innovation [87] identified a collagen-derived peptide that interacts with integrin α4, promoting organized scar formation, improving vascular perfusion, and preserving cardiac function post-MI. Maintaining a balance between collagen and elastin in the ECM is critical for proper cardiac repair. MI-induced remodeling often results in increased collagen and reduced elastin, leading to impaired elasticity and heightened stiffness [88,89]. Biomaterials that combine both proteins, such as collagen–elastin scaffolds, offer synergistic benefits—collagen supports cell adhesion, while elastin imparts resilience. Matriderm, a bovine-derived collagen–elastin matrix, is a clinical example used in dermal applications, enhancing vascularization and reducing scar formation [90]. Experimental work with electrospun collagen–elastin scaffolds has shown improved mechanical strength, fibroblast proliferation, and angiogenesis [91]. In bone regeneration, such scaffolds have facilitated osteoblast differentiation and matrix mineralization [92].

Collagen also serves as a delivery system for regenerative therapies. Encapsulating stem cells in collagen matrices enhances cell engraftment, survival, and differentiation into cardiomyocytes and endothelial cells, leading to improved cardiac regeneration and reduced fibrosis [93]. Furthermore, Clift et al. (2021) [94] employed matrix-assisted laser desorption/ionization (MALDI) to trace collagen hydrogel-derived peptides within infarcted myocardium, revealing their integration into host ECM and their post-translational modifications such as hydroxyproline, essential for collagen stability. Despite these advancements, challenges remain in translating collagen-based therapies to the clinic. Material strength, long-term performance, and scalability need to be optimized. Nevertheless, the diverse structural and bioactive properties of collagen—and its engineered forms—make it a promising platform for myocardial repair and fibrosis modulation.

## 7. Dietary Intervention of Collagen Supplements in Cardiac Ischemia

Several studies reported dietary supplements contribute to better health outcomes, such as fewer complications, shorter hospital stays, lower readmission rates, and lower mortality rates [95,96,97]. It helps prevent and manage chronic diseases like obesity, diabetes and cardiovascular disease by addressing poor dietary patterns and fostering healthy eating habits. It also supports recovery and improves quality of life [98]. Collagens are abundant in fish and meat, and so it is often utilized as a nutritional supplement. However, its oral absorption is limited, it must be hydrolyzed before it becomes a physiologically accessible supplement [99].

**Figure 4:**
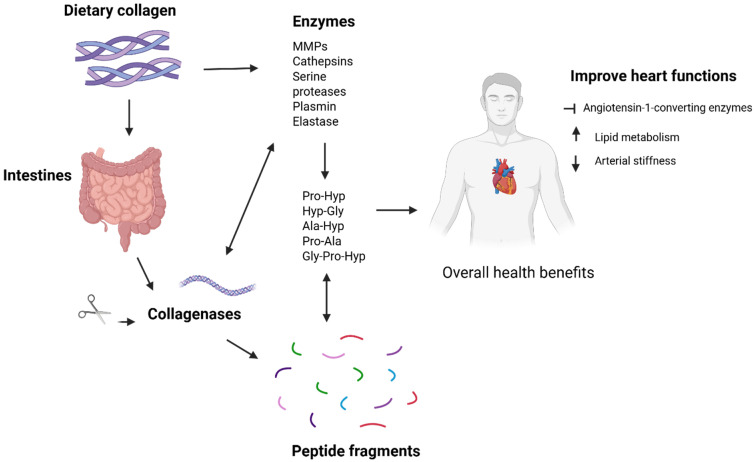
A Schematic diagram showing how dietary collagen is degraded in the body and exerts beneficial benefits (Illustration made using BioRender; https://BioRender.com/bcfc0ao, accessed on 21 August 2025).

Collagen peptides have recently gained popularity as a functional ingredient in the food sector. The physiological effects of circulating dipeptides highlight the relevance of consuming collagen peptides [100]. Collagen tripeptide (CTP), an abundant tripeptide, was shown to help maintain the flexibility of artery walls. Specifically, CTP has demonstrated promise in lowering arterial stiffness, a crucial contributor in atherosclerosis and cardiovascular illnesses, by affecting low density lipoprotein (LDL) and high-density lipoprotein (HDL) levels (Figure 4). This stiffness, which is frequently associated with atherosclerosis and LDL/HDL cholesterol abnormalities, obstructs the natural flow of blood through arteries, increasing the risk of heart disease [101]. Collagen peptides play important biological roles, such as inhibiting angiotensin I-converting enzyme activity [102], acting as signal messengers in anabolic cellular processes in cartilage, tendons, and ligaments [103] and activating the mechanistic target of rapamycin signaling pathway [104]. Furthermore, collagen peptides may improve lipid metabolism and insulin sensitivity (Wang et al., 2008) [105] decrease Cytochrome p450, nitric oxide, and prostaglandin, and increase bradykinin [106]. Over the last decade, several clinical studies have focused on the benefits of collagen peptide supplementation (CPS) on cardiovascular disease (CVD)-related indicators; however, these studies have yielded inconsistent results [107]. Zdzieblik et al. (2021) [107] found that 15 g of particular CPS per day for 12 weeks significantly enhanced fat-free mass and decreased fat mass in middle-aged males. Furthermore, Kouguchi et al. (2013) [108] revealed that 2.9 g/day of chicken collagen hydrolysate supplementation for 12 weeks significantly lowered SBP and DBP in patients with moderate hypertension. Another randomized controlled research found that daily supplementation with 2 g of CPS for 12 weeks did not affect body mass, fat mass, LDL, HDL, TAG, total cholesterol, or fasting blood sugar in healthy volunteers [109]. In addition, Jendricke et al. (2020) [110] found that 15 g of particular CPS per day for 12 weeks dramatically boosted fat-free mass while having no effect on body mass or fat mass in active women. As a result, further data is required to validate and contribute to the actual effect of CPS.

## 8. Current Research and Applications

To date, a variety of biomaterials with suitable physical and chemical characteristics have been developed for the treatment of CI. These include synthetic and natural polymeric biomaterials, such as fibrin, collagen, alginate, chitosan, hyaluronic acid, decellularized extracellular matrix tissues, and matrigel [111,112]. As the most prevalent ECM protein, collagen has emerged as a viable biomaterial option for heart regeneration and engineering. Collagen-based biomaterials can improve the transport of bioactive molecules for cardiac repair and regeneration, improve stem cell adhesion, retention, and engraftment, and temporarily mechanically support the ischemic heart. In addition, many researchers have identified that collagen-based biomaterials possess a large surface area which can integrate with stem cells, growth factors, and other components [113,114]. The right materials for heart tissue should have the following essential characteristics: For these materials to endure the substantial volume shift that occurs during heartbeat, they need to be compliant and mechanically robust [115]. To preserve remaining healthy cardiomyocytes and transplanted cells, they should replicate the natural myocardial milieu and three-dimensional structure to support cellular functions [116]. A manufactured cardiac engineering material should also be non-immunogenic, electro-physiologically stable, degradable, and highly porous with numerous interconnected pores [117,118].

In conclusion, collagen has emerged as a highly promising biocompatible material for the treatment of CI due to its unique chemical, mechanical, and structural characteristics. Collagen-based materials can be classified as either synthetic or naturally sourced [59]. The latter are composed of ECM from mammalian sources that can be isolated from the small intestine, bladder, liver, skin, skeletal muscle, heart valves, blood vessels, and so forth for clinical applications of naturally sourced collagen [12,55]. ECM biomaterials eliminate the cellular components that can lead to immunogenicity and retain a significant amount of the natural biochemical and biological cues following a sequence of biochemical, mechanical, and physical processes, making it an excellent material for tissue engineering [12,119].

Several pre-clinical studies have demonstrated the potential of collagen-based therapies in cardiac repair. A study found that injecting type I collagen directly into the myocardium could slow the progressive decline of heart function. The ejection fraction (EF) of rats recovered by 8.2% in the group treated with type I collagen [120]. Our lab has shown that compared to vehicle MI controls, long-term treatment post-MI with a type I collagen peptide promotes a two-fold improvement in EF in a severe model of permanent occlusion [87]. Serpooshan et al. (2013) [121] discovered that a collagen patch stably adhered to the surface of both the healthy myocardium and the infarcted zone, without eliciting further immune/inflammatory response. Immunostaining and histological analysis revealed the migration of several natural cardiac cells into the patch, including cardiac fibroblasts, epicardial-derived progenitor cells, α-actinin+ cardiomyocytes, and smooth muscle cells. Furthermore, they discovered that hearts treated with the collagen patch had considerably enhanced EF and fractional shortening (FS) and inhibited maladaptive cardiac remodeling, resulting in restricted fibrosis and decreased dilation of the left ventricular chamber [121]. Fibrosis is driven by persistent pro-inflammatory and pro-fibrotic signaling (e.g., TGF-β1, angiotensin II) that causes excessive, disorganized collagen deposition by activated myofibroblasts. Collagen-based biomaterials differ in that they are structurally organized to mimic healthy ECM, biodegradable and integrated into the host’s-controlled repair cycle, and lack chronic injury signals that drive pathological collagen overproduction [122]. Generally, collagen-based biomaterials are engineered to mimic physiological ECM signals, guiding the wound-healing process toward organized tissue repair rather than excessive, disordered collagen deposition.

Another study examined vehicle, collagen matrix alone, and collagen-chitosan injectable hydrogel as treatments for MI [123]. They discovered that the collagen-chitosan matrix inhibited adverse ventricular remodeling by increasing MMP-9/tissue inhibitor of metalloproteinase 2 ratio, which conferred normal ECM homeostasis and limited cardiac fibroblast-to-myofibroblast differentiation, resulting in greater left ventricular mass preservation and less fibrosis. The collagen-chitosan matrix significantly reduced macrophage infiltration, with CD68+ cells (a surface marker for macrophages) being less prevalent in the MI heart than in other groups. More crucially, they showed that the collagen-chitosan matrix improved heart function. Over a 3-week follow-up period, mice treated with collagen-chitosan matrix showed significant improvements in EF and FS, as well as arteriole density. However, the study found that using a collagen hydrogel as a stand-alone therapy did not slow the loss of heart function in MI mice, contradicting prior research [113]. The disparity could be related to variances in animal models, delivery routes, or delivery time points.

Additionally, studies have investigated the idea of replacing the lost myocardium with stem cell transplantation. Clinical and animal studies have shown that this strategy is both safe and practical [117,124]. Different types of stem cells, such as Embryonic Stem Cells (ESCs), Induced Pluripotent Stem Cells (iPSCs), Mesenchymal Stem Cells (MSCs), Adipose-Derived Stem Cells (ADSCs), and Cardiac Stem Cells (CSCs), have been employed to treat MI thus far. However, very low acute retention and engraftment of cells due to the ischemia environment, inflammation, immunological response, mechanical washout of cells from the constantly beating heart, and flushing by the coronary arteries limit the use of stem cell therapy [125,126,127]. Skin collagen-based biomaterials have been extensively investigated as cell delivery vehicles for various stem cell populations to get around these restrictions and improve the therapeutic outcome of cardiac stem cell transplantation [128].

Collagen matrices have been shown by [129] to promote cardiomyoblasts’ (immature heart muscle cells that can be transplanted into damaged heart tissue to help repair it) early survival following transplantation into infarct myocardium. H9C2 cardio-myoblasts expressing green fluorescent protein reporter and firefly luciferase were implanted into the infarcted area together with collagen matrix. Additionally, on days 5 (534  ±  115 vs. 219  ±  34) and 8 (274  ±  34 vs. 180  ±  23), optical bioluminescence imaging revealed that bioluminescence signals were considerably higher in the H9c2/collagen group compared to the H9c2/PBS group. Furthermore, the results of histological analysis and immunohistochemistry were identical [129]. In a different study, Roche et al. (2014) [130] examined four different biomaterial delivery methods for delivering human MSCs to the infarct border zone: two injectable hydrogels (alginate, chitosan/β-glycerophosphate) and two epicardial patches (alginate, collagen). With no discernible difference between the individual biomaterials from time 0 to 24 h (average of 50–62% for biomaterials, 9% for saline control), they showed that all four biomaterials enhanced the acute retention of stem cells in the infarcted heart and that cell retention in all four biomaterials treated MI was better than that of the saline control [130]. Nevertheless, it is unclear how collagen-based biomaterials encourage cell adherence and proliferation. Enhancing the effectiveness of stem cell therapy may involve creating a favorable milieu, increasing the expression of cell adhesion molecules, or giving cells unique cell binding sites [131,132].

Collagen therapy offers a wide range of medicinal and cosmetic applications, owing to its ability to provide structure, strength, and support to many tissues. As previously stated, it’s utilized for tissue healing and regeneration; others include cosmetic operations like Collagen Induction Therapy, and even in commercial uses like leather and gelatin manufacture. However, collagen-based therapies can have limitations, including rapid degradation in vivo, immune response depending on the collagen source, and limited mechanical strength in high-pressure environments like the heart. To overcome these, researchers are to explore more cross-linking techniques (e.g., genipin, EDC/NHS) for enhanced durability, hybrid biomaterials (collagen with elastin, alginate, or synthetic polymers), and bioactive molecule incorporation for targeted cell signaling. The future of collagen-based cardiovascular healing lies in personalized, multifunctional scaffolds capable of guiding tissue regeneration while integrating safely into the host myocardium.

## 9. Conclusions

In conclusion, collagen is prevalent in the body, and improper deposition or processing is related to a variety of illnesses. This review focuses on the advancements and potential for the use of collagen-based therapies in cardiovascular disease. Collagen is being researched in a range of disorders at both the preclinical and clinical phases. Studies on both humans and animals have shown that collagen-based biomaterials hold a lot of promise for cardiac ischemia intervention. The biocompatibility, porosity, biodegradability, ease of purification and availability, non-immunogenicity, and electrophysiological stability of collagen are among its many desirable qualities for heart regeneration. Collagen-based biomaterials have already been used as a stem cell delivery system to improve engraftment, adhesion, and retention in recent decades. Furthermore, growth factors, cytokines, and stem cell-mobilizing factors are among the bioactive substances that collagen-based biomaterials can prolong in the infarcted area, improving cardiac function.

In the future, we believe that the convergence of technology developments in cardiac biology and personalized medicine will lead to better knowledge of how collagen can be targeted for regenerative medicine applications. Notably, the exact process by which collagen-based biomaterials, including cellular components, treat MI is still unknown. There are several options, such as supplying mechanical stability, encouraging angiogenesis, developing into transplanted cells’ functioning cardiomyocytes, and using a paracrine mechanism. Paracrine signaling may be the most crucial of these processes for treating MI, and collagen-based biomaterials can enhance stem cell survival by giving cells a transient supporting ECM, which eventually raises the levels of favorable paracrine signals. In addition, the ideal distribution route and duration for collagen-based biomaterials are still unknown. With further research, collagen-based biomaterials have great potential for cardiac tissue engineering and regeneration.

## Figures and Tables

**Table 1:** Collagen types, key features, location, category and corresponding references. Collagens that comprise cardiovascular tissues are bolded.

Types of Collagens	Key Features	Location	Category/References
**I**	Accounts for around 60% of the cardiovascular collagens. Composed of two α1(I) chains and one α2(I) chain in a triple helix structure. High content of glycine, proline, and hydroxyproline for stability. Highly cross-linked, giving tensile strength	Skin, tendon, bone, ligaments, **interstitial tissues**	Fibrillar collagen/[13]
II	Accounts for 60–75% in the vitreous collagen fibrils. Rich in hydroxylysine and hydroxyproline, contributing to stability. Highly glycosylated compared to type I.	Intervertebral disc, cartilage, vitreous humor	Fibrillar collagen/[14]
**III**	Accounts for around 30% of the cardiovascular collagens. Composed of **three identical α1(III) chains** arranged in a triple helix. Often co-distributed with type I collagen in a 1:2 or 1:3 ratio, forming interwoven fibers.	**Vessels**, uterus, skin, **muscle**	Fibrillar collagen/[13].
**IV**	Accounts for <10% of cardiovascular collagens. localized to the endothelium basement membrane and the basement membranes of smooth muscle cells of the intima and media.	Found in various tissues, including the skin, kidneys, lungs and **blood vessels**.	Network forming Collagen/[15,16].
V	A fibrillar collagen that helps organize and stabilize type I collagen fibrils in the ECM. Key features include its ability to bind to other collagens like types I, III, and XI	Human placenta and dermis. Associated with type I.	Fibrillar collagen/[17,18].
**VI**	Less than 10% of cardiovascular collagens. Prevalent in the media and adventitia of arteries, in thin connective tissue septa, in the area surrounding capillaries, and in the endomysium next to myocardial cells.	Widely distributed in various tissues, including skeletal muscle, skin, lung, **blood vessels**, cornea, intervertebral discs, peripheral nerves, brain, myocardium, and adipose tissue.	Beaded filament-forming collagens/[19,20]
VII	A non-fibrillar collagen, major component of anchoring fibrils, connects the epidermis and its underlying basement membrane to the papillary dermis.	Majorly found in the skin	Anchoring fibril Collagens/[21]
**VIII**	Assemble diverse patterns of networks. Descemet’s membrane is a significant component of the basement membrane of corneal endothelial cells (EC).	Found in **endothelial cells**, keratinocytes, mast cells, **microvascular endothelial cells**, and some tumor cells. It is also found on a number of extracellular matrices, such as sclera, skin, and glomerulus.	Network-Forming Collagens/[22]
X	Composed of three identical α1(X) chains encoded by the COL10A1 gene. Relatively short triple-helical domain compared to fibrillar collagens. Forms hexagonal lattice networks in specialized extracellular matrices	Hypertrophic chondrocytes, growth plates and fracture callus	Network-Forming Collagens/[23]
XI	Similar in size to collagen types I and II. Fibril-forming collagen, a key component of cartilage, specifically involved in the organization and stability of type II collagen fibrils	Broadly distributed in articular cartilage, testis, trachea, tendons, trabecular bone, skeletal muscle, placenta, lung, and the brain	Fibrillar Collagens/[24]
**XV**	Contains multiple interruptions in its triple-helical domain. Has a large N-terminal non-collagenous (NC) region and a C-terminal globular domain. It represents <10% of the vascular collagen	**Basement membrane of the vascular wall**	FACIT collagens/[25]
XVIII	Present in three isoforms generated by alternative promoters and splicing. Composed of a collagenous triple-helical region with multiple interruptions and large non-collagenous (NC) domains. The C-terminal NC1 domain contains endostatin, a potent anti-angiogenic peptide. Represents less than 10% of vascular collagens.	Primarily found within basement membranes throughout the body, particularly in epithelial and endothelial tissues	Non-fibrilla Collagens/[26]

## Data Availability

Not applicable.

## Abbreviations

The following abbreviations are used in this manuscript:

CI	Cardiac Ischemia
ECM	Extracellular Matrix
FACITs	Fibril-associated collagens with interrupted triple helices
AGE	Advanced glycation end product
LOX	Lysyl oxidase
TEVAR	Thoracic endovascular aortic repair
EVAR	Endovascular aneurysm repair
VEGF	Vascular endothelial growth factor
EDC/NHS	EDC (1-ethyl-3-(3-dimethylaminopropyl) carbodiimide hydrochloride) and NHS (N-hydroxysuccinimide)
ESCs	Embryonic Stem Cells Induced
iPSCs	Pluripotent Stem Cells
MSCs	Mesenchymal Stem Cells
ADSCs	Adipose-Derived Stem Cells
CSCs	Cardiac Stem Cells
bFGF	basic Fibroblast Growth Factor
UV	Ultraviolet
DDRs	Discoidin domain receptors
MMPs	Matrix metalloproteinases
HUVECs	Human Umbilical Vein Endothelial Cells
PlGF	Placental growth factor
PLGA	poly(lactic-co-glycolic acid)
MALDI	Matrix-assisted laser desorption/ionization
CTP	Collagen tripeptide
CPS	Collagen peptide supplementation
LDL	Low density lipoprotein
HDL	High density lipoprotein
EF	Ejection fraction
FS	Fraction shortening
